# Evidence of *d*-phenylglycine as delivering tool for improving *l*-dopa absorption

**DOI:** 10.1186/1423-0127-17-71

**Published:** 2010-09-06

**Authors:** Chun-Li Wang, Yang-Bin Fan, Hsiao-Hwa Lu, Tung-Hu Tsai, Ming-Cheng Tsai, Hui-Po Wang

**Affiliations:** 1 Taipei Medical University College of Pharmacy, 250 Wu-Hsing St., Taipei, 110-31, Taiwan; 2 Roche Products Ltd., Taipei, Taiwan; 3 Institute of Traditional Medicine, School of Medicine, National Yang-Ming University, 155 Li-Nong Street, Section 2, Taipei, Taiwan; 4 Department of Pharmacology, College of Medicine, National Taiwan University, No. 1, Section 1, Jen-Ai Rd., Taipei, Taiwan

## Abstract

**Background:**

*l*-Dopa has been used for Parkinson's disease management for a long time. However, its wide variety in the rate and the extent of absorption remained challenge in designing suitable therapeutic regime. We report here a design of using *d*-phenylglycine to guard *l*-dopa for better absorption in the intestine via intestinal peptide transporter I (PepT1).

**Methods:**

*d*-Phenylglycine was chemically attached on *l*-dopa to form *d*-phenylglycine-*l*-dopa as a dipeptide prodrug of *l*-dopa. The cross-membrane transport of this dipeptide and *l*-dopa via PepT1 was compared in brush-boarder membrane vesicle (BBMV) prepared from rat intestine. The intestinal absorption was compared by *in situ *jejunal perfusion in rats. The pharmacokinetics after i.v. and p.o. administration of both compounds were also compared in Wistar rats. The striatal dopamine released after i.v. administration of *d*-phenylglycine-*l*-dopa was collected by brain microdialysis and monitored by HPLC. Anti-Parkinsonism effect was determined by counting the rotation of 6-OHDA-treated unilateral striatal lesioned rats elicited rotation with (+)-methamphetamine (MA).

**Results:**

The BBMV uptake of *d*-phenylglycine-*l*-dopa was inhibited by Gly-Pro, Gly-Phe and cephradine, the typical PepT1 substrates, but not by amino acids Phe or *l*-dopa. The cross-membrane permeability (Pm*) determined in rat jejunal perfusion of *d*-phenylglycine-*l*-dopa was higher than that of *l*-dopa (2.58 ± 0.14 vs. 0.94 ± 0.10). The oral bioavailability of *d*-phenylglycine-*l*-dopa was 31.7 times higher than that of *l-*dopa in rats. A sustained releasing profile of striatal dopamine was demonstrated after i. v. injection of *d*-phenylglycine-*l*-dopa (50 mg/kg), indicated that *d*-phenylglycine-*l*-dopa might be a prodrug of dopamine. *d*-Phenylglycine-*l*-dopa was more efficient than *l-*dopa in lowering the rotation of unilateral striatal lesioned rats (19.1 ± 1.7% vs. 9.9 ± 1.4%).

**Conclusion:**

The BBMV uptake studies indicated that *d*-phenylglycine facilitated the transport of *l*-dopa through the intestinal PepT1 transporter. The higher jejunal permeability and the improved systemic bioavailability of *d-*phenylglycine-*l*-dopa in comparison to that of *l*-dopa suggested that *d-*phenylglycine is an effective delivery tool for improving the oral absorption of drugs like *l*-dopa with unsatisfactory pharmacokinetics. The gradual release of dopamine in brain striatum rendered this dipeptide as a potential dopamine sustained-releasing prodrug.

## Background

**Figure 1:**
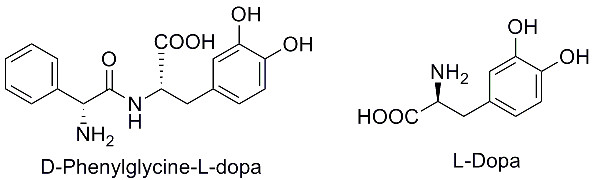
**The structures of *d*-phenylglycine-*l*-dopa and *l*-dopa**.

*l*-Dopa (Figure 1), a dopamingenic precursor, has long been used for the treatment of Parkinson's disease [1-4]. Clinically use of this drug was reported to have wide range of inter- and intra-patient variations in the rate and the extent of absorption [5,6]. The inconsistent pharmacokinetics remained as the major issue in designing optimal regime in the disease management [7,8]. The variation in oral bioavailability due to the interaction of *l*-dopa with diet protein is, in part, attributed to its complicated absorption through the amino acid transport systems [9-11]. Although many dopamine agonists emerged, *l*-dopa in combination with metabolic enzyme inhibitors is still the first choice for the treatment of Parkinson's disease [2,3].

Recent reports indicated that intestinal PepT1, a member of proton-coupled oligopeptide transporter system, is responsible for the absorption of a variety of di- and tripeptide mimetic drugs such as amino-β-lactams [12-14] and ACE inhibitors [15]. The structure feature of PepT1 substrates was established [16-18] and the transport system has been used in the design of novel oral-absorbable drugs [19,20].

Based on the thought that *d*-phenylglycine is the common moiety in the molecules of PepT1-mediated orally absorbable amino-β-lactams [21], we thought that this moiety might be useful as a seeing-eye dog for guiding *l*-dopa to transport through the intestine via PepT1. We therefore synthesized a series of *d*-phenylglycine-containing di- and tripeptide derivatives as dopamine prodrug [22]. Rationale behind the design of these compounds was that the oral bioavailability of *l*-dopa might be improved due to the affinity of *d*-phenylglycine to PepT1. Besides, the fast decarboxylation of *l*-dopa in peripheral circulation might be prevented or prolonged as the free amino group of *l*-dopa is blocked by *d*-phenylglycine.

This report describes the transport of *d*-phenylglycine-*l*-dopa via PepT1 by measuring the uptake in brush-border membrane vesicles (BBMV) prepared from rat intestine. The intestinal absorption of this compound and *l*-dopa was compared by measuring the steady-state plasma concentration after *in situ *jejunal perfusion and by determining the pharmacokinetics after oral administration in rats. Anti-Parkinsonism effects after oral administration of *d*-phenylglycine-*l*-dopa and *l*-dopa were also compared by measuring the change of the (+)-methamphetamine induced rotation of dopamine-depleted unilateral striatal-lesioned rats. Correlation between pharmacological activity and the pharmacokinetic profile was analyzed.

## Methods

### Materials

Chemicals, reagent grade for synthesis and analytical grade for biological studies, were from Sigma-Aldrich (St. Louis, MO, U.S.A), E. Merck KG (Darmstadt, Germany), Fluka Chemika (Buchs, Switzerland), Acros (NJ, U.S.A) and Wako (Richmond, VA, U.S.A) companies. Acid-washed alumina was purchased from RiedaL-de Haen Company (Spring Valley, CA, U.S.A.). Melting points were determined in Buchi (Flawil, Switzerland) 510 capillary melting point apparatus and were uncorrected. IR spectra were carried out on a Perkin-Elmer (Shelton, CT, U.S.A) 1760 FT-IR instrument. ^1^H NMR spectra were determined on a Bruker (Wissem-bourg, France) 80 MHz or Bruker 400 MHz spectrometer with chemical shifts recorded in parts per million relative to tetramethylsilane. Mass and high-resolution mass (HRMS) were measured on Finnigan (San Jose, CA. U.S.A.) MAT 4510 and JEOL (Boston, MA, U.S.A.) JNS-D300 spectrometer respectively. Branson (Danbury, CT. U.S.A.) Sonifier 450 sonicator, Kubota (Tokyo, Japan) 2010 or Eppendorf AG (Hamberg, Germany) 5415C centrifuge Model 905 incubator (Cherng Huei Instrument Co., Tainan, Taiwan) and Ystral (Ballrechten-Dottingen, Germany) Laboratory series × 10/20 homogenizer were used in the preparation of intestinal mucosal suspension. Osmolarity of test solutions was determined with Wescor 5500 vapor pressure osmometer (Wescor Company, Logan, UT, U.S.A.). Male Wistar rats (300 - 350 g) from the Animal center of National Taiwan University were used in preparing intestinal mucosal suspension, BBMV and in perfusion studies. The same species of rats weighing 180 - 200 g were used in rotational behaviour studies. Male Sprague-Dawley rats (280 - 320 g) were used for determining brain dopamine. Animal studies were in accordance with the National Institute of Health Guide for the Care and Use of Laboratory Animals.

### Brush-Boarder Membrane Vesicle (BBMV) Uptake

The intestinal cross-membrane transport of *d*-phenylglycine-*l*-dopa and *l*-dopa was investigated using simulated intestinal brush-boarder membrane vesicle [23,24]. BBMV was prepared using magnesium precipitation method [25]. Protein content was determined. The purity of BBMV was determined by measuring the activity of the marker enzymes, alkaline phosphatase and aminopeptidase. Generally, these two enzymes were enriched 8 - 21 folds in the preparation. The activity of Na^+^, K^+^-ATPase, the marker enzyme of basolateral membrane, was very small. Normal function of BBMV was confirmed by measuring the uptake of *d*-glucose. In the presence of Na^+ ^gradient ([Na^+^]_in _< [Na^+^]_out_), an overshoot phenomenon of glucose uptake with peak values of 9-11 times the equilibrium was routinely observed. The membrane vesicles were preloaded in the buffer solution containing 300 m*M *mannitol and 16 m*M *HEPES/Tris (pH 7.4) before the experiment. The uptake of test compounds in BBMV was measured by rapid filtration.

### Degradation of Compounds in Intestinal Mucosa Suspension

Mucosa suspension was prepared from the intestine of male Wistar rats according to established method [26] and was stored in an ice bath before use.

### In Situ Rat Perfusion

Literature procedure was followed for the preparation of perfusion solutions and the jejunal segments [27]. To maximize the absorption and to prevent the test compounds from being oxidized during perfusion, the experiments were performed at pH 6.0 with 0.02% (w/v) ascorbic acid added as antioxidant and nitrogen gas was bubbled through for 10 min before each experiment. Perfusion solution was pumped through the jejunal segment at a flow rate of 0.2 ml/min by a syringe pump (Stoelting, KD Scientific, U.S.A.). The jejunal segment was pre-washed with drug-free buffer for 10 min before the drug solution was pumped in. Outlet tubing samples were collected every 10 min for 6 collection periods after water and solute transport reached steady-state. The dimensionless membrane permeability Pm* [28] was measured as indications for the disappearance rate of test compound from the intestine. Plasma samples were withdrawn from carotid artery.

### Intravenous and Oral Absorption Experiments

Rats were fasted for at least 18 h prior to drug administration. Anaesthesia was induced by i.p. injection of urethane (0.15 g/100 g body weight). The rats were put under a heating lamp to maintain body temperature.

### Chromatography and Validation of Assay Methods

The HPLC system used in the assay of biological samples consisted of an autosampler (AS950, Jasco, Tokyo, Japan), a Waters Model 600E solvent delivery pump (Millipore, Milford, MA, USA), a Model LC-4C electrochemical detector with a glassy-carbon electrode (Bioanalytical Systems, Inc., West Lafayette, IN, USA), and an integrator (Macintosh LC II with Macintegrator I). A Nucleosil^® ^10 SA cationic ion-exchange column (10 μm, 300 × 4.0 mm, Macherey-Nagel, Düren, Germany) with a mobile phase comprising NaCl (50 m*M*) and Na_2_-EDTA (1.0 m*M*) in 0.1 *M *ammonium phosphate buffer (pH 2.0) at a flow rate of 2.0 ml/min was used for the elution of the samples. The detection limits of *d*-phenylglycine-*l*-dopa and *l*-dopa were 50 ng/ml and 25 ng/ml, respectively. HPLC assay methods were validated by determining the precision and accuracy of intra-day and inter-day analysis of serum standards over a period of 6 days. The coefficients of variation for inter- and intraday assays were less than 15% for both compounds (n = 6).

### Pharmacokinetic Analysis

According to the literature [29,30], the area under the plasma concentration-time profile (AUC) was calculated by log-linear trapezoidal rule. Plasma concentration after i.v. administration of drugs were also fitted to a non-compartment model using PCNONLIN and Akeike's Information Criteria, sum of squared residuals, residual plot and correlation coefficient were use for determination of the compartment model. The residual area after the last observed data point was calculated as C_last_/k, where C_last _is the last observed concentration, and k is the corresponding terminal rate constant. Terminal half-life (t_1/2_) was estimated compartment model-independently. The fraction of absorption was calculated according to Equation 1.

(1) $$\text{BA}=\frac{\frac{{\text{AUC}}_{\text{oral}}\cdot {\text{k}}_{\text{oral}}}{{\text{dose}}_{\text{oral}}}}{\frac{{\text{AUC}}_{\text{iv}}\cdot {\text{k}}_{\text{iv}}}{{\text{dose}}_{\text{iv}}}}\times 100\%$$

### Brain Microdialysis

Single dose *d*-phenylglycine-*l*-dopa (50 mg/kg in 2.5 mL of normal saline) was administered i. v. via femoral vein to anesthetized male Sprague-Dawley rats (280 - 320 g). The body temperature of the rats was maintained at 37°C with a heating pad throughout the experiment. The rat was immobilized in a stereotaxic frame (David Kopf Instruments, Tujunga, CA, USA), the skull was surgically exposed, and a hole was drilled with a trephine into the skull based on stereotaxic coordinates. The brain microdialysis system consisted of a CMA/100 microinjection pump (CMA, Stockholm, Sweden) and a microdialysis probe. The dialysis probes (3 mm in length) were made of silica capillary in a concentric design with their tips covered by dialysis membrane (Spectrum, 150 μm outer diameter with a cut-off at nominal molecular mass of 13000, Laguna Hills, CA, USA). The probe was placed into right striatum (0.2 mm anterior to bregma and 3.2 mm lateral to midline) and perfused with Ringer's solution (147 mM Na+; 2.2 mM Ca++; 4 mM K+; pH 7.0) at a flow-rate of 1 μl min^-1^. The position of each probe was verified at the end of experiments. The dialysates was collected at 10 min intervals and aliquots of 10 μl was assayed by microbore HPLC.

The HPLC system consisted of a pump (BAS PM-80, West Lafayette, IN, USA) and an on-line injector (CMA 160, Stockholm, Sweden) equipped with a 10 μl sample loop, a reversed phase C18 microbore column (particle size 5 μm, 150 × 1 mm I.D.; Bioanalytical Systems, West Lafayette, IN, USA) and an EC detector (BAS-4C amperometric) coupled to a glassy carbon working electrode and referenced to a Ag/AgCl electrode at 750 mV with a range set at 50 nanoamper. Output data from the detector were integrated via an EZChrom chromatographic data system (Scientific Software, San Ramon, CA, USA). The mobile phase for analyzing striatal dopamine, eluted at a flow rate of 0.05 ml/min, comprised 80 ml acetonitrile, 2.2 m*M *sodium 1-octanesulfonate, 14.7 m*M *monosodium dihydrogen orthophosphate, 30 mM sodium citrate, 0.027 m*M *EDTA, and 1 ml diethylamine in one liter double distilled water, adjusted to pH 3.5 by orthosphoric acid (85%). The elute was filtered through a Millipore 0.22 μm filter and degassed prior to use.

### Rotational Behavior of Rats[31-34]

Male Wistar rats (180 - 200 g) were anesthetized with pentobarbital sodium (30 mg/kg body weight, i.p.) and the heads were fixed in a DaviD-Kopf steric taxic frame. A solution of 6-hydroxydopamine (6-OHDA, 2.00 mg/ml × 8 ml) in saline was infused using Paxinos and Watson coordinates (AP 5.3, L 2.0, H 7.8 mm, [34]) into the unilateral substantia nigra compacta (SNc) of brain with a syringe pump through a 30 gauge stainless steel needle at a flow rate of 2 μl/min. After two weeks of recovery period, the 6-OHDA treated rats were placed in a spherical bowl (radius 20 cm) and secured by a thoracic harness which was connected to a 486 PC computer for automatic recording of rotation induced by (+)-methamphetamine (MA). The rotational behavior of rats was recorded 10 min after MA treatment (MA in saline, 4.00 body weight mg/kg of rat, s.c.). The numbers of turns recorded were defined as the control value (T_0_) for each individual animal. Only animals showing a T_0 _greater than 400 were chosen for further experiments. After two weeks of a wash-out period the animals were subjected to drug treatment. Single dose (0.051 mmol) of each test compound was administered orally to rats 5 min prior to MA treatment (4.00 mg/kg body weight, s.c.). The rotation counted for a period of 110 min starting 10 min after MA treatment was recorded as T_d _for each tested rat. The percentage of reduction in rotation for each animal was calculated and presented as (T_d _- T_0_)/T_0 _× 100%.

### Data Analysis

Data analysis were performed on Visual dBase and SPSS/PC+ and were represented as mean ± SE for n experiments. Treatment differences were evaluated by paired-t test.

## Results

### *d*-Phenylglycine-*l*-dopa Uptake in BBMV

**Figure 2:**
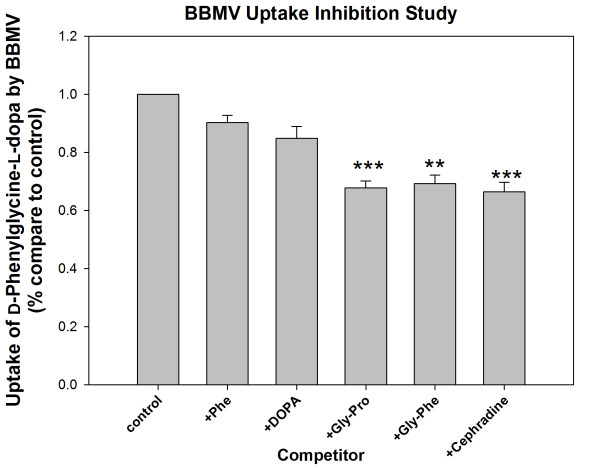
The uptake of *d*-phenylglycine-*l*-dopa in BBMV with or without the presence of *l*-Phe, *l*-dopa, *l*-Gly-*l*-Pro, *l*-Gly-*l*-Phe and cephradine (**: *p *< 0.01; ***: *p *< 0.001.). The BBMV was prepared according to material and methods. The BBMV preparation (20 ml containing approximately 20 mg protein/ml) was added into 200 ml of a reaction buffer (composed of 300 m*M *mannitol, 25 m*M *HEPES/Tris buffer pH 7.4, (pH was adjusted by adding MES) and the test solution (to 1 - 2 m*M *of final conc.) was added. After incubation at room temperature for acquired time, an ice-cold stop solution (1.5 ml) containing NaCl (150 m*M*) and HEPES/Tris (16 m*M*, pH 7.4) was added and the solution was filtered through a filter paper (Whatman WCN, 0.45 μm pore size, 2.5 cm diameter) under a vacuum. The filter paper was washed twice with 3 ml of the same stop solution. The test compound remained on the filter paper were extracted with 0.5 ml of 0.01 *M *aqueous HCl solution by virtue of a vortex motion. The solution (100 μl) was injected onto the HPLC column. Test compound bound on the filter paper was determined for correction in different runs using preparations without BBMV added.

The uptake of *d*-phenylglycine-*l*-dopa in BBMV was measured. Amino acid *l*-Phe or *l*-dopa, dipeptide *l*-Gly-*l*-Pro or *l*-Gly-*l*-Phe, or cephradine was added for investigating the competition with *d*-phenylglycine-*l*-dopa in BBMV uptake (Figure 2).

### Stability of *d*-Phenylglycine-*l*-dopa in Intestinal Mucosal Suspension

**Figure 3:**
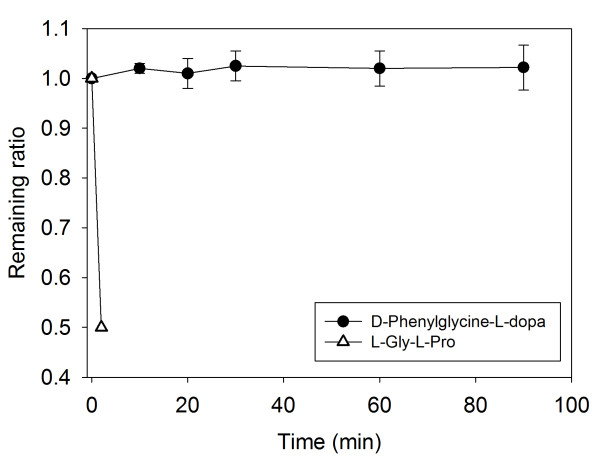
**Comparison of the stability of *d*-phenylglycine-*l-*dopa and *l*-Gly-*l*-Phe in rat intestinal mucosa suspension**. Each point represents mean ± SE. of 3 experiments. A methanolic solution (100 μl) of the test compound (1 mg/ml) was diluted with an isotonic mannitol buffer solution (pH 6.5, 2.4 ml) as the stock solution. This stock solution (1 ml) was mixed with the freshly prepared mucosal suspension (1 ml). The mixture was incubated in a water bath at 37°C and subjected to sampling at intervals between zero to 90 min of incubation. Each sampled solution (200 μl) was denatured with 0.8 ml of MeOH and centrifuged at 6,600 *g *for 5 min. Each of the supernatant (20 - 100 μl) was subjected to HPLC assay.

The stability of *d*-phenylglycine-*l*-dopa in the intestine was determined prior to the intestinal absorption studies. In order to simulate intestinal microclimate pH, the compound was incubated with the intestinal mucosal suspension in a pH 6.5 isotonic buffer solution. *l*-Gly-*l*-Phe comprising essential amino acids degraded rapidly with only 50% of recovery after 2 min of incubation. *d*-Phenylglycine-*l*-dopa, on the other hand, was very stable with almost 100% of recovery after 90 min of incubation (Figure 3).

### Permeability of *d*-Phenylglycine-*l*-dopa in Rat Intestine

The absorption of *d*-phenylglycine-*l*-dopa and *l*-dopa was compared in rats by *in situ *single-pass jejunal perfusion experiments. Amidon's dimensionless cross-membrane permeability (P_m_*) was determined as a parameter of intestinal absorption [28]. The steady-state plasma concentration was also determined (Table 1).

**Table 1:** Plasma concentrations of *d*-phenylglycine-*l*-dopa and *l*-dopa measured in *in situ *single-pass jejunal perfusion experiments.

Compound	No. of Experiments	Pm*	Blood concentration (μg/ml)	Molar ratio of blood concentration (μg/ml)
*d*-phenylglycine-*l*-dopa	4	2.58 ± 0.14	64.6 ± 5.40	31.10
*l*-dopa	3	0.94 ± 0.10	1.24^a^	1.02

^a ^*l*-dopa was detected only in the plasma sample from one of the three rats tested. It was below detection limit in plasma samples of the other two rats. Data presented are mean ± SD of n experiments.

### Pharmacokinetic Profile in Rats

**Figure 4:**
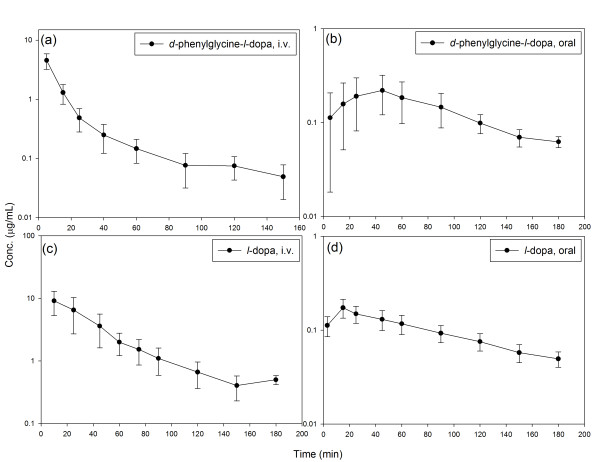
**Plasma concentration-time profile of *d*-phenyglycine-*l*-dopa (a), (b) and *l*-dopa (c), (d) after i.v. (a), (c) and oral (b), (d) administration in Wistar rats (n = 6)**. The aqueous solution of test compound with dose equivalent to 5.97 mg/kg body weight of *l*-dopa was administered either intravenously from the tail vein or orally by a feeding tube. Blood samples were collected from the carotid artery at time intervals of from 1 to 180 min. Heparin sodium (25 I.U./ml in 0.3 ml of saline) was added to blood samples, and were then centrifuged at 6,600 *g *for 5 min. Plasma was stored at -78°C until being analyzed. A 200 μl of the plasma sample in a 10 ml test tube was mixed with 500 μl of 1.0 *M *Tris buffer (pH 8.6, adjusted by EDTA-2Na^+^) and 10 μl of 3,4-dihydroxybenzylamine (DHBA, 2 μg/ml) was added as internal standard. Alumina 100 mg was then added and then shake for 15 sec and the supernatant was decanted. The alumina was washed four times with 5 ml of water, and the adsorbed compounds on the alumina was eluted with 200 μl of an acidic solution (0.9 ml of glacial acetic acid in 4.0 ml of 1.0 *M *phosphate buffer). A 30 μl of the eluent was then analyzed by HPLC.

**Figure 5:**
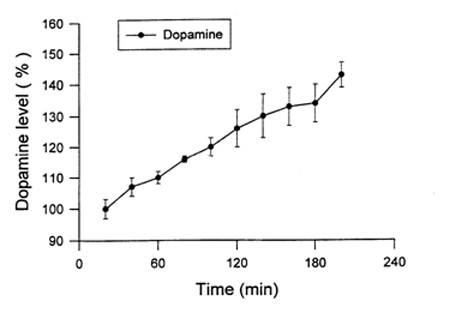
**Striatal dopamine level after i. v. injection of *d*-phenylglycine-*l*-dopa**. Values represent the group mean ± s.e.m. (n = 4). Single dose *d*-phenylglycine-*l*-dopa (50 mg/kg in 2.5 mL of normal saline) was administered i.v. via femoral vein to anesthetized male Sprague-Dawley rats (280 - 320 g). The dialysates collected from the brain microdialysis probe was subjected to HPLC to measure the striatal dopamine concentration at 10 min interval.

The mean plasma concentration-time profiles after single dose oral and i.v. administration of *d*-phenylglycine-*l*-dopa and *l*-dopa are depicted in Figure 4. The pharmacokinetic parameters calculated with the data of plasma concentration-time curves based on the non-compartmental model analysis were summarized in Table 2. The fraction of oral absorption (BA) was calculated according to Equation 1. The Striatal dopamine level after i.v. injection of *d*-phenylglycine-*l*-dopa (50 mg/kg) is depicted in Figure 5.

**Table 2:** Pharmacokinetic parameters derived from non-compartmental analysis after i.v. and oral administration of *d*-phenylglycine-*l*-dopa and *l*-dopa in rats (mean ± SD, n = 6).

	*d*-phenylglycine-*l*-dopa		*l*-dopa	
	**i.v**.	Oral	**i.v**.	Oral
AUC (mg·min/ml)	62.53 ± 19.68	28.85 ± 8.52	459.81 ± 195.14	27.37 ± 4.60
t_1/2_(min)	254.10 ± 73.05	142.50 ± 23.71	101.52 ± 27.74	184.80 ± 46.20
Cl_p _(l/kg/min)	0.18 ± 0.06	0.29 ± 0.10	0.02 ± 0.02	0.01 ± 0.00
Vd_ss _(l/kg)	11.01 ± 5.08	35.7 ± 17.1	1.22 ± 0.89	1.22 ± 0.36
t_max_(min)	--	38.30 ± 17.72	--	25.02 ± 16.10
Fraction of absorption (%)	--	27.58 ± 4.56	--	0.87 ± 0.24

### Anti-Parkinsonism Activity

The *in vivo *anti-Parkinsonism effect was determined with conventional rotation model measured in 6-OHDA-treated unilateral striatal-lesioned rats elicited rotation with (+)-methamphetamine (MA) [32,35]. As shown in Table 3, *d*-phenylglycine-*l*-dopa as well as *l*-dopa demonstrated inhibition of MA-induced rotation of rats. With equal molar of test compound administered, the activity of *d*-phenylglycine-*l*-dopa in reducing the rotation of rats was significantly higher than that of *l*-dopa.

**Table 3:** Percent reduction of (+)-methamphetamine-induced rotation in unilateral nigrostriatal-lesioned rats after oral administration of *d*-phenylglycine-*l*-dopa and *l*-dopa.

Compound	dose (mg/kg)	no. of experiment	Reduction in rotation (mean ± SE %)
*d*-phenylglycine-*l*-dopa	16.7	6	19.1 ± 1.7*
*l*-dopa	10.0	6	9.9 ± 1.4

***(*p *< 0.001)

## Discussion

The BBMV uptake of *d*-phenylglycine-*l*-dopa was significantly inhibited by dipeptides *l*-Gly-*l*-Pro (***p < 0.001), *l*-Gly-*l*-Phe (**p < 0.01) and cephradine, a typical PepT1 substrate (*** *p *< 0.001), while was less inhibited by *l*-Phe and *l*-dopa, suggesting that PepT1 might be involved in the uptake of this dipeptide. We previously reported a kinetic study on the BBMV uptake of *d*-phenylglycine-α-methyldopa. The uptake of this dipeptide was also significantly inhibited by typical PepT1 substrate [22]. The high value of Michaelis-Menten kinetic parameter (Vmax/Km) in comparison to that of passive diffusion (Kd) at low concentrations suggested that PepT1 dominates the transport of the *d*-phenylglycine-containing dipeptide through the intestine. Both results indicated that *d*-phenylglycine increased the intestinal transport of amino acid α-methyldopa and *l*-dopa via PepT1.

The absorption of oral drugs in human can be evaluated as dimensionless permeability P_m_* in *in situ *single-pass perfusion in rats despite the complicated process of absorption in the gastrointestinal tract [28,36,37]. The high P_m_* demonstrated by *d*-phenylglycine-*l*-dopa (2.58 ± 0.14) in comparison to that of *l*-dopa (0.94 ± 0.10) indicated the high absorption of this dipeptide in the intestine. The steady-state plasma concentration of *d*-phenylglycine-*l*-dopa after the perfusion was 31.1 fold, in terms of molar ratio, higher than that of *l*-dopa, indicated that this dipeptide was better absorbed than *l*-dopa.

The pharmacokinetic profiles upon i.v. and oral administration of *d*-phenylglycine-*l*-dopa and *l*-dopa were compared. Although the volume of distribution after i.v. injection of *d*-phenylglycine-*l*-dopa was higher than that of *l*-dopa, this dipeptide was cleared much faster than *l*-dopa from the plasma. This made the systemic bioavailability (AUC) of *d*-phenylglycine-*l*-dopa 7 times lower than that of *l*-dopa (62.53 ± 19.68 vs. 459.81 ± 195.14 mg·min/ml). On the contrary, the AUC of *d*-phenylglycine-*l*-dopa was comparable to that of *l*-dopa upon oral administration (28.85 ± 8.52 vs. 27.37 ± 4.60 mg·min/ml). As a result, the fraction of oral absorption of *d*-phenylglycine-*l*-dopa was 31 fold higher than that of *l*-dopa (27.58 ± 4.56% vs. 0.87 ± 0.24%).

The striatal dopamine level increased gradually after i.v. injection of *d*-phenylglycine-*l*-dopa and had not reached plateau 3.5 hours when the anaesthetized mice woke up. The gradual release of dopamine in brain striatum rendered this dipeptide as a dopamine sustained-releasing prodrug.

*d*-Phenylglycine-*l*-dopa after oral administration demonstrated higher activity than *l*-dopa in reducing the MA-induced rotation in rats with statistical significance (19.1 ± 1.7% vs. 9.9 ± 1.4%, ***p < 0.001), suggesting its anti-Parkinsonism activity. Whether the activity came from the dipeptide *per se *or from the released dopamine needs further investigation. Correlation between the pharmacological activity and the pharmacokinetic parameters indicated that the high activity demonstrated by *d*-phenylglycine-*l*-dopa might partially come from its better oral absorption.

## Conclusion

*d*-Phenylglycine-*l*-dopa was proved to be better absorbed from the intestine than *l*-dopa. The BBMV uptake suggested that *d*-phenylglycine might act as a seeing-eye dog for guiding *l*-dopa to transport through the intestine via intestinal PepT1 oligopeptide transporter. The higher anti-Parkinsonism activity of this dipeptide in comparison to that of *l*-dopa might come from the improved oral bioavailability. The pharmacokinetic profile of striatial dopamine indicated that *d*-phenylglycine *l*-dopa might be useful as a slow dopamine-releasing prodrug for therapeutic use. The improved intestinal permeability with improved oral bioavailability as a consequence, suggested the potential use of *d*-phenylglycine as an effective delivery tool for drugs with unsatisfied oral absorption.

## Abbreviations

PepT1: Intestinal peptide transporter T1; BBMV: Brush-boarder membrane vesicle; MA: Methamphetamine

## Competing interests

The authors declare that they have no competing interests.

## Authors' contributions

CLW and YBF carried out PK and rat rotational studies and drafted the manuscript. HHL carried out permeability studies. MCT designed rat rotational behavior studies. THT carried out the brain microdialysis studies. HPW conceived and is responsible for the study. All authors read and approved the final manuscript.
